# Investigating antimicrobial-resistant bacteria from exotic domestic birds – a One Health concern

**DOI:** 10.29374/2527-2179.bjvm001624

**Published:** 2024-08-03

**Authors:** Bianca da Costa Tavares da Silva, Daniel Ubriaco Oliveira Gonçalves de Carvalho, Victoria Tiemi Sorbello Sakauchi, José Soares Ferreira, Adriana Cortez, Marcos Bryan Heinemann, Natália Carrillo Gaeta

**Affiliations:** 1 Veterinarian, Departamento de Medicina Veterinária Preventiva e Saúde Animal, Faculdade de Medicina Veterinária e Zootecnia, Universidade de São Paulo (USP). São Paulo, SP, Brazil.; 2 Veterinarian, Curso de Medicina Veterinária, Universidade de Guarulhos (UnG), Guarulho, SP, Brazil; 3 Veterinarian, Universidade Santo Amaro (UNISA), Santo Amaro, SP, Brazil; 4 Veterinarian, Curso de Medicina Veterinária, Faculdades Integradas Campos Salles (FICS), São Paulo, SP, Brazil

**Keywords:** antibiotic, surveillance, One Health, cockatiels, budgie, canary, antibiótico, vigilância, Saúde Única, calopsitas, periquito, canário

## Abstract

Antimicrobial resistance is a natural mechanism in microorganisms, making the treatment of infections more complex in human and veterinary medicine. Global exotic and ornamental bird markets have significantly increased, and the close relationship between pets and humans makes exploring the potential role of these birds as vectors for the spread of antimicrobial-resistant bacteria imperative. This study aimed to use culture-dependent methods to investigate cloacal bacteria and the presence of antibiotic-resistant bacteria in four breeding stocks of ornamental birds. Cloacal swab samples were collected from 53 birds (canaries = 32, cockatiels = 17, and budgies = 4) and used for culturing and isolating facultative anaerobic and/or obligatory aerobic Gram-positive and Gram-negative bacteria. The antimicrobial susceptibility profile of each isolate was determined by the disk diffusion method. Thirty-four isolates were obtained, most of which belonged to the *Staphylococcus* genus. Bacterial richness was higher in canaries and in one of the breeding stockings, where Gram-negative bacteria were more abundant than in the others. In addition, canaries exhibited a predominance of resistant isolates, particularly multidrug-resistant strains, probably due to prophylactic antimicrobial usage. Most Gram-negative bacteria were resistant to at least one drug tested. A vancomycin-resistant *Enterococcus faecalis* strain was isolated. Most *Staphylococcus* strains were resistant to gentamycin, followed by penicillin. Eight strains were cefoxitin-resistant, including oxacillin-resistant *S. epidermidis*, in which the *mecA* gene was detected. Understanding the prevalence of resistance in avian species is crucial in the collaborative pursuit of maintaining antibiotic effectiveness and strengthening public health defense against emerging infectious risks.

## Resumo

A resistência antimicrobiana é um mecanismo natural dos microrganismos, complicando o tratamento de infecções na medicina humana e veterinária. O mercado global de aves exóticas e ornamentais cresceu significativamente, e a relação próxima entre esses animais e humanos destaca a necessidade de investigar o papel das aves na disseminação de bactérias resistentes. Este estudo utilizou métodos dependentes de cultura para examinar bactérias cloacais e a presença de resistência a antibióticos em quatro plantéis de aves ornamentais. Amostras de suabe cloacal foram coletadas de 53 aves (canários = 32, calopsitas = 17, periquitos = 4) e usadas para cultivar e isolar bactérias Gram-positivas e Gram-negativas, facultativas anaeróbias e aeróbias obrigatórias. A suscetibilidade antimicrobiana foi determinada pelo método de difusão em disco. Foram obtidos 34 isolados, principalmente do gênero *Staphylococcus*. A riqueza bacteriana foi maior nos canários e em um dos plantéis, onde houve aumento de Gram-negativos. Canários mostraram predominância de isolados resistentes, especialmente cepas multirresistentes, provavelmente devido ao uso profilático de antimicrobianos. A maioria das bactérias Gram-negativas foi resistente a pelo menos um fármaco testado. Um *Enterococcus faecalis* resistente à vancomicina foi isolado. A maioria dos *Staphylococcus* foi resistente à gentamicina e penicilina; oito cepas foram resistentes à cefoxitina, incluindo *S. epidermidis* resistente à oxacilina com o gene *mecA* detectado. Compreender a prevalência de resistência em aves é crucial para manter a eficácia dos antibióticos e fortalecer a saúde pública contra riscos infecciosos emergentes.

## Introduction

Antimicrobial resistance is a natural mechanism in microorganisms (Prestinaci et al., 2015). This refers to the evolving changes over time that render bacteria, viruses, fungi, and parasites less susceptible to the effects of medications. The mechanisms underlying resistance can be either intrinsic or acquired, with the latter being associated with genetic mutations and/or horizontal gene transfer (Prestinaci et al., 2015). This resistance makes the treatment of infections more complex, amplifying the threat of disease transmission, severe illness, and even death (World Health Organization, 2021).

Resistant bacteria can traverse the boundaries between humans and animals, particularly in close relationships between guardians and their pets. Human methicillin-resistant *Staphylococcus aureus* (MRSA) clones have been identified in dogs and cats (Botoni et al., 2016; Scherer et al., 2018). Moreover, instances of resistant bacteria found in poultry have been documented in parrots, including Congo parrots, budgies, and cockatiels (Lamb et al., 2014). These findings emphasize the potential for the interplay of antibiotic-resistant strains across human-animal interfaces.

The global exotic and ornamental bird market has significantly increased as more families opt for smaller companion animals owing to smaller households and limited available time. In 2016, an estimated population of around 39 million domesticated birds was reported in Brazil, with cockatiels emerging as the preferred species (Guedes, 2016). As the exotic and ornamental bird industry grows, it is essential to investigate whether these birds can spread antimicrobial-resistant bacteria, which is important in regard to human and animal medicine as part of the One Health framework. This study aimed to investigate antimicrobial-resistant and cloacal aerobic bacteria in exotic domestic birds using a culture-dependent technique.

## Materials and methods

A total of 53 exotic and ornamental birds (6 months to two years), including young and adult males and females, were selected for random evaluation from four commercial breeding sites located in São Paulo City (Brazil), designated A (N = 22), B (N = 9), C (N = 15), and D (N = 7) (Table 1). Specifically, 32 cloacal swab samples were collected from domestic canaries (*S. canaria domestica*), 17 from cockatiels (*N. hollandicus*), and four (4) from budgerigars (*M. undulatus*). These species are considered exotic domestic animals by the Brazilian Institute of Environment and Natural Resources (IBAMA) (Brazil, 1998, 2019); therefore, this research did not require authorization from the Biodiversity Authorization and Information System (SISBIO).

**Table 1:** Distribution of the samples collected by each stock and host species.

Stock	Host	Sample collected % (N/T)
A	Canary	54.5 (12/22)
	Cockatiel	45.5 (10/22)
		
*B*	Cockatiel	100 (7/7)
		
*C*	Canary	100 (15/15)
		
*D*	Canary	55.5 (5/9)
	Budgie	44.5 (4/9)
		
Total		100 (53/53)

During their juvenile phase, these birds are typically provided with artificial alimentation using commercial products tailored to their respective species. Throughout their lifespans, these birds were given unrestricted access to water. All of the animals were housed in cages designed with appropriate dimensions and animal density. Daily care and handling were consistently performed by the same employees, thereby minimizing stress levels and contributing to the promotion and maintenance of overall well-being.

We compiled a comprehensive profile of each breeding site by gathering information on (i) the use of antibiotics, and (ii) breeders' knowledge of antimicrobial-resistant bacteria and the One Health concept. Breeders were interviewed regarding several aspects, including (a) prophylactic and metaphylactic application of antimicrobials, (b) specific medications employed to combat infections and whether a veterinarian prescribed them, (c) sources from which they obtained these medications, (d) their observations on any decline in the efficacy of antimicrobials over time, (e) their perceptions of the quantity and the types of side effects associated with antibiotic misuse in poultry, (f) their awareness of bacterial resistance and the One Health approach, and (g) their views on their potential role in preventing antimicrobial resistance and their understanding of that role.

At the time of sample collection, all animals were clinically assessed by a simple clinical examination, including general aspects, activity of the chest musculature, and cleanliness of the cloaca. This confirmed their health status. All birds had to be clinically healthly prior to sampling in order to avoid interference with the results. None of the animals developed diarrhea before the sampling. The cages were cleaned daily and covered with newspaper sheets. Cleanliness was confirmed by visual inspection.

Cloacal fecal samples were obtained from each bird by introducing a sterile swab into the cloaca and rotating it for 15 s. The swabs were placed in sterile tubes and refrigerated until they were transported to the laboratory. The swabs were placed in three milliliters of buffered peptone water (Difco, USA) and incubated at 37º C for 18–24 h.

### Culture and isolation of Gram-negative and Gram-positive bacteria

Enterobacteria were investigated by plating 10 µl of peptone water growth in MacConkey agar plates (Acumedia®, USA). To culture and isolate *Salmonella* spp., all of the samples were streaked on Xylose Lactose Tergitol 4 (XLT4) agar (Difco, USA). Gram-positive bacteria were cultured and isolated by plating 10 µL of the peptone water growth in sheep blood agar. Plates were incubated at 35º C±2º C for 24 hours. Colonies were identified using matrix-assisted laser desorption ionization time-of-flight mass spectrometry (MALDI-TOF MS).

### Antimicrobial susceptibility test and gene detection

Antimicrobial susceptibility profiles were evaluated using the disk diffusion test (Kirby-Bauer method) according to the M100 document from the Clinical and Laboratory Standards Institute (2022). For Gram-negative (GN) bacteria, the following drugs (DME, Brazil) and their respective concentrations in micrograms were used: aztreonam 30 (ATM), trimethoprim-sulfamethoxazole 1.25/23.75 (SUT), ceftriaxone 30 (CRO), Nalidixic Acid 30 (NAL), ceftazidime 30 (CAZ), amoxicillin with clavulanic acid 30 (AMC), cefotaxime 30 (CTX), cefoxitin 30 (CFO), cefepime 30 (CPM), ciprofloxacin 05 (CIP), imipenem 10 (IMP), ertapenem 10 (ERT), meropenem 10 (MPM), tetracycline 30 (TET), and gentamicin 10 (GEN). For *Staphylococcus* spp., penicillin 10 (PEN), gentamicin 10 (GEN), tetracycline 30 (TET), ciprofloxacin 05 (CIP), enrofloxacin 10 (ENO), sulfazotrim 25 (SUT), cefoxitin 30 (CFO), oxacillin 30 (OXA), and chloramphenicol 30 (CLO) were used. Finally, penicillin 10 (PEN), vancomycin 30 (VAN), ciprofloxacin 05 (CIP), and chloramphenicol 30 (CLO) were tested against *Enterococcus* spp. In addition to the phenotypic profile, the presence of important resistance genes such as *mecA*, *mecC*, *blaZ*, and *vanA* was evaluated using PCR. These conditions were described by Mehrotra et al. (2000), Paterson et al. (2014), Martineau et al. (2000), and Dutka-Malen et al. (1995).

### Data analysis

Relative and absolute frequencies demonstrate the results. Shannon and Simpson diversity indices for bird species and breeding sites were calculated using the "vegan" package v.2.6.4. Figures were generated using the ggplot2 package v.3.3.3 (https://ggplot2.tidyverse.org). All figures and calculations were generated using R Studio v.4.0.5 (RStudio Team, 2020).

## Results

### Breeding stock profile

The data regarding antimicrobial usage collected from all breeding stocks indicated that antibiotics were administered exclusively upon a veterinarian's prescription. All breeders mentioned purchasing antibiotics from human or veterinary pharmacies. Moreover, in Area A, antimicrobial treatment was halted once the animals displayed signs of improvement when sick, with the actual duration of treatment varying depending on the individual animals. Additionally, nestling birds in group C received prophylactic antibiotics, particularly cefotaxime, for a duration of seven days. Only breeder B demonstrated awareness of the One Health concept among the breeders. Finally, every breeder was aware that the improper use of antimicrobials could lead to issues by either fostering the development of bacterial resistance or creating a perception that treating a sick animal would be challenging.

### Gram-positive and Gram-negative isolated bacteria

Thirty-four isolates were obtained from 53 cloacal swab samples, most came from canaries (67.6%; 23/34), followed by cockatiels (17.6%; 6/34), and budgies (14.7%; 5/34). Regardless of the bird type, most isolates belonged to the *Staphylococcus* genus. *S. kloosi, S. delphini, S. aureus*, and *S. epidermidis* were particularly common. Furthermore, canaries were the primary source of most enterobacteria isolates, including clinically significant species such as *E. coli* and *K. pneumoniae*. *Enterococcus* spp., an essential GP genus, was also consistently isolated from all bird species (Table 2).

**Table 2:** Frequency of Gram-positive, and Gram-negative bacteria isolated from the cloacal swab samples from canaries, cockatiels and budgies.

**Bacteria**	**Canaries % (N/T)**	**Cockatiels % (N/T)**	**Budgies % (N/T)**
*Staphylococcus*	62.5 (15/23)	50.0 (03/06)	40.0 (02/05)
*S. kloosii*	40.0 (06/15)	-	-
*S. delphini*	20.0 (03/15)	-	-
*S. aureus*	13.3 (02/15)	-	-
*S. epidermidis*	13.3 (02/15)	-	-
*S. warnery*	7.6 (01/15)	-	50.0 (01/02)
*S. capitis*	7.6 (01/15)	-	-
*S. saprophyticus*	-	33.3 (01/03)	-
*S. xylosus*	-	33.3 (01/03)	-
*S. haemolyticus*	-	33.3 (01/03)	50.0 (01/02)
			
*Enterobacteriaceae*	30.4 (07/23)	16.7 (01/06)	20 (01/05)
*E. cloacae*	28.6 (02/07)	-	-
*S. maltophilia*	14.3 (01/07)	-	-
*A. pitti*	14.3 (01/07)	-	-
*A. radioresistens*	-	100 (01/01)	-
*C. freundii*	14.3 (01/07)	-	-
*K. oxytoca*	14.3 (01/07)	-	-
*K. pneumoniae*	14.3 (01/07)	-	-
*E. coli*	-	-	100 (01/01)
			
Other Gram-positive	4.3 (01/23)	33.3 (02/06)	40.0 (02/05)
*E. faecalis*	100 (01/01)	50 (01/02)	100 (02/02)
*E. hirae*	-	50 (01/02)	-

**Figure 1:**
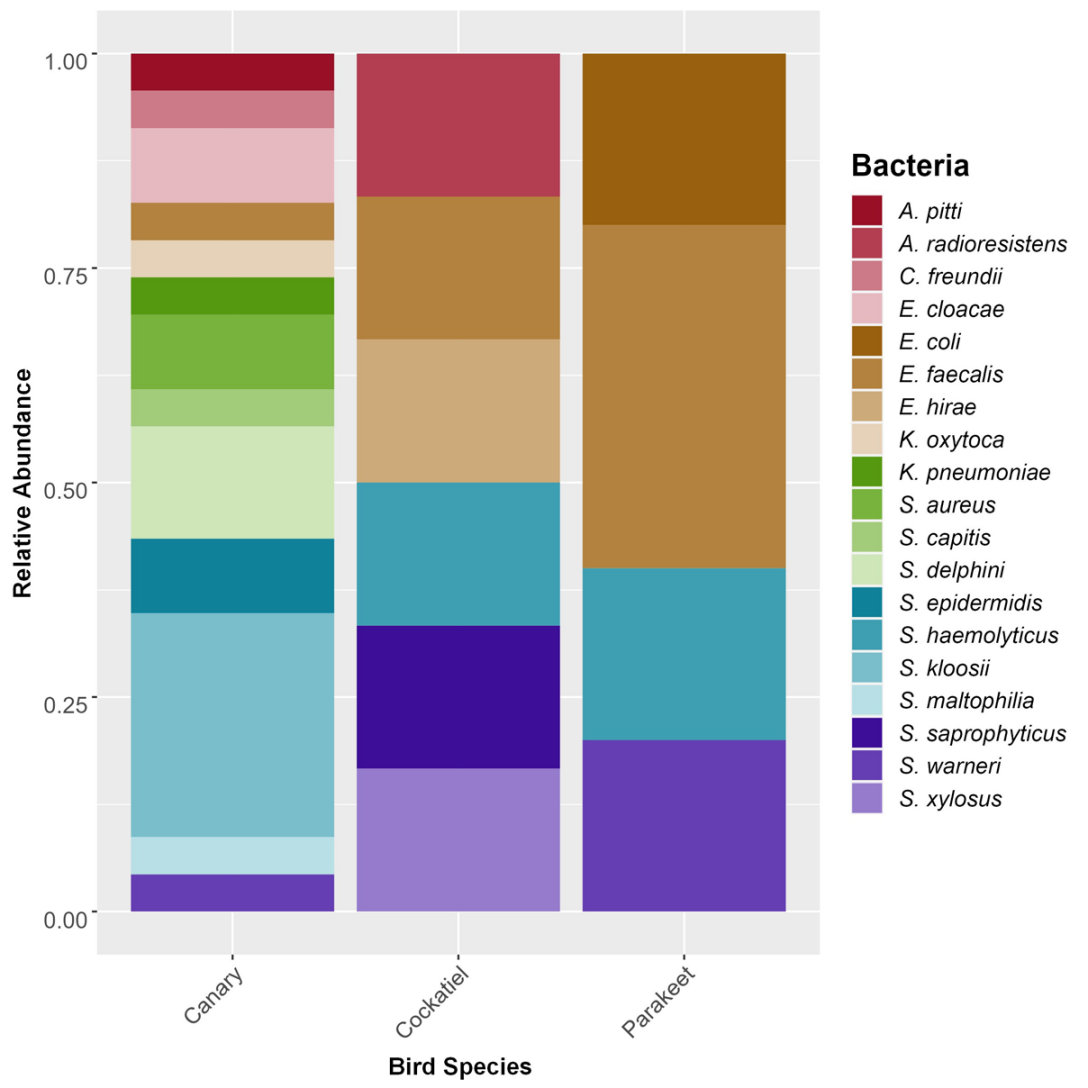
Relative abundance of the bacteria isolated from the cloacal swab samples of exotic domestic birds, according to avian species.

The richness of bacteria isolated from the cloaca of the birds studied differed. For example, canaries (N = 13) had a higher richness than cockatiels (N = 6) and budgets (N = 4) (*P* = 0.003), as shown in Figure 1. Despite this variation in species richness, no statistically significant differences were observed in the Shannon and Simpson diversity indices (*P* = 0.36), which could be attributed to the fact that both diversity indices consider the number of species and their evenness. GP richness differed among bird species (*P* = 0.01), particularly between canaries (N = 7) and budgies (N = 3), possibly because of the limited number of budgies enrolled in the study.

**Figure 2:**
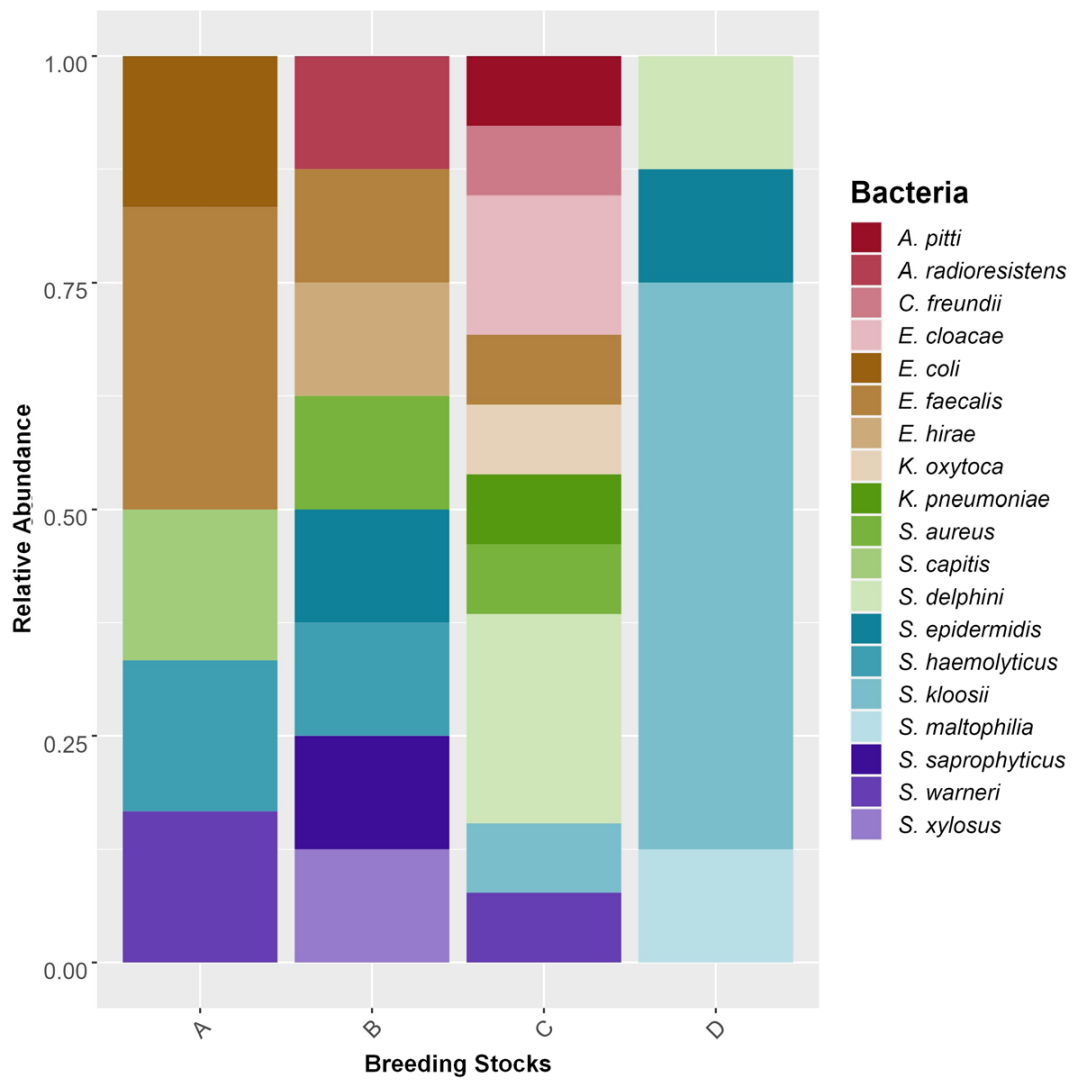
Relative abundance of the bacteria isolated from the cloacal swab samples of exotic domestic birds according to the breeding stocks.

Concerning breeding sites, breeding stock C had the highest number of isolated bacteria (N = 10, *P* = 0.74), as shown in Figure 2. Additionally, breeding stock C contained more Gram-negative species (N = 5) than the other breeding stocks, where only one such species was observed (*P* = 0.65). It is worth mentioning that no statistically significant differences were observed in the Shannon and Simpson diversity indices (*P* = 0.39). Finally, interestingly, no Gram-negative bacteria were isolated from breeding stock D, from which only *Staphylococcus* spp. were isolated.

### Antimicrobial susceptibility and genetic determinants

**Figure 3:**
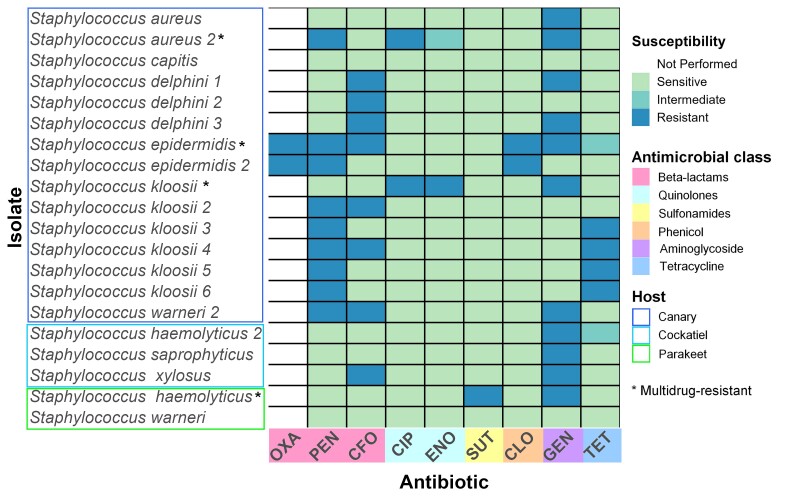
Antimicrobial susceptibility profile of *Staphylococcus* species isolated from the cloacal swab samples of exotic domestic birds.

Fifteen drugs were tested against GN strains, and it was found that eight of the isolates (88.9%; 8/9) were not susceptible to at least one of the drugs. A varied antimicrobial susceptibility profile was observed across the different bacterial groups, regardless of the host. In addition to intrinsic resistance, most isolates in this group were also resistant to tetracycline, aztreonam, ceftriaxone, ceftazidime, and nalidixic acid, and 22.9% of them were resistant to ceftriaxone (Table 2). Most of the five *Enterococcus* strains tested were resistant to at least one drug (80%; 4/5), and two were vancomycin-resistant (*vanA* gene not detected) and were classified as multidrug-resistant (MDR) (Table 3). Finally, regarding *Staphylococcus*, a majority exhibited resistance to gentamicin, followed by penicillin, cefoxitin, tetracycline, ciprofloxacin, chloramphenicol, enrofloxacin, and sulfamethoxazole-trimethoprim (Figure 3). The *blaZ* gene (related to penicillin resistance) was detected in 45% (9/20) of the penicillin-resistant isolates. In addition, both strains of *S. epidermidis* were resistant to oxacillin, in which the *mecA* gene was detected. Eight other *Staphylococcus* spp. strains (40%; 08/20) were cefoxitin-resistant; however, the *mecA* gene was not detected. The *mecC* gene was not detected in any oxacillin/cefoxitin-resistant isolates. Two strains (10%, 2/20) were multidrug-resistant.

**Table 3:** Antimicrobial susceptibility of the bacteria isolated from budgies, cockatiels and parakeets according the breeding stock from São Paulo, Brazil.

Stock	Bacteria	Sensitive	Intermediate	Resistant
A	*E. coli*	ATM, SUT, ERT, CRO, NAL, CFO, CTX, AMC, CAZ, CIP, CPM, MPM, TET	IPM	GEN
	*K. pneumoniae*	ATM, SUT, ERT, IPM, MPM, CRO, NAL, CFO, CTX, AMC, CAZ, CIP, CPM, GEN, TET	-	-
	*E. faecalis*	CIP, TET, PEN, VAN, CLO	-	-
	*E. faecalis*	PEN, VAN, CLO	-	CIP, TET
	*S. capitis*	SUT, CFO, CIP, GEN, TET, PEN, OXA, CLO, ENO	-	-
	*S. haemolyticus*	SUT, CFO, CIP, GEN, TET, PEN, OXA, ENO	CLO	-
	*S. warneri*	SUT, CFO, CIP, GEN, TET, CLO, ENO	-	PEN
B	*A. radioresistens*	SUT, ERT, IPM, MPM, NAL, CFO, AMC, CAZ, CIP, CPM, GEN	ATM, CRO, CTX	TET
	*E. faecalis*	TET, PEN, VAN, CLO	-	CIP
	*E. faecalis*	PEN	-	CIP, TET, VAN, CLO
	*S. aureus*	SUT, CFO, CIP, GEN, TET, CLO, ENO	-	OXA, PEN
	*S. epidermidis*	SUT, CIP, ENO	CLO	CFO, GEN, TET, PEN, OXA
	*S. saprophyticus*	SUT, CFO, CFO, GEN, TET, CLO, ENO	-	PEN, OXA
	*S. xylosus*	SUT, CFO, CIP, GEN, PEN, CLO, ENO	-	TET, OXA
	*S. haemolyticus*	CFO, CIP, GEN, TET, CLO, ENO	-	SUT, PEN, OXA
C	*A. pitti*	SUT, NAL, AMC, IPM, CIP, MPM, GEN, TET	ATM, ERT, CFO, CTX, CAZ, CPM	CRO
	*K. oxytoca*	SUT, ERT, CRO, CFO, CTX, AMC, IPM, CIP, CPM, MPM, GEN	-	ATM, NAL, CAZ, TET
	*C. freundii*	ATM, SUT, ERT, CRO, CTX, CAZ, IPM, CIP, CPM, MPM, GEN, TET	AMC	NAL, CFO
	*E. cloacae*	SUT, NAL, AMC, IPM, CIP, CPM, MPM, GEN, TET	CFO, CTX, CAZ	ATM, ERT, CRO
	*E. cloacae*	ATM, SUT, ERT, MPM, IPM, CRO, NAL, CFO, CTX, AMC, CAZ, CIP, CPM, GEN, TET	-	-
	*E. faecalis*	GEN	CLO	CIP, TET, PEN
	*S. kloosii*	SUT, CFO, GEN, TET, CLO	-	CIP, PEN, OXA, ENO
	*S. delphini*	SUT, CFO, CIP, GEN, PEN, CLO, ENO	-	TET, OXA
	*S. delphini*	SUT, CFO, CIP, GEN, PEN, CLO, ENO	-	TET
	*S. delphini*	SUT, CFO, CIP, GEN, PEN, CLO, ENO	-	TET, OXA
	*S. aureus*	SUT, CFO, TET, CLO	ENO	CIP, GEN, PEN, OXA
	*S. warneri*	SUT, CFO, CIP, CLO, ENO	-	GEN, TET, PEN, OXA
D	*S. maltophilia*	SUT, NAL, CIP, GEN	-	ATM, ERT, IPM, MPM, CRO, CFO, CTX, AMC, CAZ, CPM, TET
	*S. epidermidis*	SUT, CFO, CIP, GEN, TET, CLO, ENO	-	PEN, OXA
	*S. kloosii*	SUT, CIP, GEN, TET, CLO, ENO	-	CFO, PEN, OXA
	*S. kloosii*	SUT, CFO, CIP, GEN, CLO, ENO	-	TET, PEN, OXA
	*S. kloosii*	SUT, CFO, CIP, GEN, CLO, ENO	-	TET, PEN, OXA
	*S. kloosii*	SUT, CIP, GEN, CLO, ENO	-	CFO, TET, PEN, OXA
	*S. kloosii*	SUT, CFO, CIP, GEN, CLO, ENO	-	TET, PEN, OXA

Canaries exhibited a predominance of resistant isolates, particularly multidrug-resistant strains. Finally, despite prophylactic treatment with cefotaxime, breeding-stock C isolates were not resistant to this drug.

## Discussion

In the present study, cloacal-cultivable aerobic bacteria from exotic domestic pets were analyzed using culture-dependent techniques. Passerines contain many GP bacteria, with *Staphylococcus* spp. being one of the most common types of cloacal bacteria (Latham et al., 2012). This information is consistent with the findings of our study. Likewise, psittacines were also found to have a more significant presence of GP bacteria in their cloaca than that in GN species (Videvall et al., 2018). In our study, GN bacteria were isolated from the cloacal samples of psittacines; however, it is crucial to highlight that none of the animals exhibited any clinical symptoms of any disease during the sampling period. These findings underscore the importance of integrating culture and isolation data with clinical information to enhance diagnostic precision. Regarding breeding stocks, breeding stock C contained more GN species. Notably, at this specific breeding site, animals receive a 7-day prophylactic treatment (cefotaxime), which may contribute to intestinal dysbiosis and promote the proliferation of GN bacteria. Despite this treatment, stock C bacteria did not exhibit resistance to cefotaxime. Although the treatment regimen may influence the bacterial composition, the duration of treatment may not have been sufficient to promote the selection of bacteria resistant to this particular drug. In addition, cefotaxime-resistant isolates may belong to strains that were not isolated in this study. Further studies are therefore required to gain a deeper understanding of this phenomenon.

The antimicrobial susceptibilities of the isolated strains were also evaluated, revealing important results. *K. oxytoca* exhibited multidrug resistance, whereas *K. pneumoniae* was the only isolate that was sensitive to all tested drugs. Among GN bacteria, a noteworthy finding emerged in the identification of *Stenotrophomonas maltophilia*, a bacterial species renowned for its multidrug resistance (Chang et al., 2015; Rizek et al., 2018), which is primarily attributed to natural mutations facilitating the development of efflux pumps in the plasma membrane and, to a lesser extent, the creation of antimicrobial-inactivating enzymes (Ahmed & Baptiste, 2018; Gil-Gil et al., 2020). Although considered a secondary pathogen, its presence in the cloaca is significant because it may help other bacterial species acquire resistance genes through transduction and conjugation, similar to other GN drug-resistant bacteria (Brooke, 2021).

Enterococci, especially *E. faecalis* and *E. faecium*, are opportunistic bacteria associated with many hospital-acquired MDR infections in humans (Ahmed & Baptiste, 2018). The overuse of antibiotics such as subtherapeutic avoparcin in animal production has led to an increase in MDR *Enterococcus* spp. in animals and humans, including strains that are not susceptible to vancomycin (Ahmed & Baptiste, 2018; Hammerum, 2012). Notably, we identified a vancomycin-resistant *E. faecalis* (VRE) strain from a canary, underscoring the significance of the close bond between pets and their owners in the One Health context. The *vanA* operon is usually involved in vancomycin resistance in *Enterococcus* (Hollenbeck & Rice, 2012); however, the canary strain does not harbor the *vanA* gene, suggesting that other van genes may be present. Emergence of vancomycin-resistant enterococci poses a significant challenge in human medicine. Moreover, even clinically healthy individuals can harbor these bacteria (Amberpet et al., 2016) and may transfer them to their pets, including exotic domestic birds, during routine handling and interactions. However, even after the avoparcin ban, VRE have been isolated from healthy animals, including dogs. For example, Pasotto et al. (2016) evaluated the prevalence and antimicrobial resistance traits of VRE in household dogs in Italy, and their findings illustrated that dogs frequently harbor antimicrobial-resistant enterococci, emphasizing their role as reservoirs for resistant strains, including those resistant to last-line drugs. Moreover, the elevated prevalence of ciprofloxacin-resistant *Enterococcus* spp. aligns with documented trends, highlighting the persistent growth of this resistance phenomenon over the past few decades (Schaberg et al., 1992). This issue mirrors concerns observed in prescribing antibiotics for managing human urinary tract infections (Lee, 2013). Consequently, identification of resistant strains in avian populations has significant One Health implications.

*Staphylococcus* genus was the most isolated bacteria and most exhibited resistance to gentamycin, followed by penicillin and cefoxitin. Typically, resistance to penicillin in *Staphylococcus* is mediated by the *blaZ* gene, which encodes the production of a beta-lactamase (Lowy, 2003). Detection of the *blaZ* gene in 45% (9/20) of the penicillin-resistant isolates suggests the dissemination of this gene among exotic birds. *S. epidermidis* strains were resistant to oxacillin, and two isolates (10%; 2/20) were multidrug-resistant. One was a *S. aureus* isolate, a bacterium on the World Health Organization's (WHO) 2017 list of high-priority pathogens for the research and development of new antibiotics. Eight strains (40%; 08/20) were cefoxitin-resistant, including oxacillin-resistant *S. epidermidis*, for which the *mecA* gene was detected, characterizing it as a methicillin-resistant strain. The *mecC* gene was not detected in this study. Methicillin-resistant *Staphylococcus* refers to strains resistant to methicillin/oxacillin and other antibiotics within the penicillin class and cephalosporins (Alghamdi et al., 2023), which makes the treatment of infections caused by these bacteria more challenging, as it limits the available antibiotic options. Although *Staphylococcus aureus* is commonly implicated in methicillin resistance (Algammal et al., 2020), other Staphylococcus species may exhibit this resistance profile, even in exotic animals. For instance, a guinea pig with suspected pyelonephritis was recently found to host multidrug- and methicillin-resistant *S. epidermidis* (Gaeta et al., 2022). Furthermore, methicillin-resistant Staphylococci have been found in the feces of wild birds, suggesting that these birds may play a significant role in carrying and spreading resistant strains into the environment. The authors advocate ongoing surveillance of resistant bacteria in birds to better understand and manage the environmental spread of antimicrobial resistance.

It is important to note that although the isolates demonstrated additional resistance phenotypes, the primers specifically targeting the discussed genetic determinants were the only ones accessible during this research phase.

Canaries exhibited a predominance of resistant isolates, particularly multidrug-resistant isolates. The larger sample size of the studied canaries further confirmed this observation. Additionally, most of these strains originated from breeding stocks with a history of prophylactic antibiotic use, providing a plausible explanation for the observed outcomes. Tareen and Zahra (2023) recently demonstrated that the routine prophylactic use of antimicrobials in Thai pig farms is associated with increased resistance rates in commensal *Escherichia coli*.

The overuse and improper utilization of antibiotics in both healthy animals and humans fuels the escalating threat of antibiotic resistance. The WHO has encouraged actions to avoid the global spread of antimicrobial resistance, including reducing critically important antibiotics in food animals, particularly growth promoters, and prophylactic use. The WHO also emphasizes that prophylaxis should be administered to healthy animals if the disease has been diagnosed in other animals in the same group (World Health Organization, 2017).

## Conclusions

Our results highlight the potential involvement of avian species in the dissemination of drug-resistant strains. Addressing antimicrobial resistance requires a comprehensive One Health approach to mitigate antimicrobial proliferation. A thorough understanding of the prevalence of resistance in avian species is pivotal in the collective endeavor to uphold antibiotic efficacy and fortify public health defense against emerging infectious threats.
